# How Americans eat red and processed meat: an analysis of the contribution of thirteen different food groups

**DOI:** 10.1017/S1368980022000416

**Published:** 2022-05

**Authors:** Sarah M Frank, Lindsey Smith Taillie, Lindsay M Jaacks

**Affiliations:** 1 Carolina Population Center, University of North Carolina at Chapel Hill, Chapel Hill, NC, USA; 2 Department of Nutrition, Gillings School of Global Public Health, University of North Carolina at Chapel Hill, Chapel Hill, NC, USA; 3 Global Academy of Agriculture and Food Security, Alexander Robertson Building, The University of Edinburgh, Easter Bush Campus, Midlothian EH25 9RG, UK

**Keywords:** Animal-source foods, Food behaviours, Diet surveys, Environment and public health

## Abstract

**Objective:**

Dietary patterns characterised by high intake of red and processed meat are associated with detrimental health and environmental outcomes. To better understand how Americans consume red and processed meat, this study examined the food groups that are the greatest contributors to red and processed meat intake in US diets.

**Design:**

Cross-sectional analysis of total red and processed meat, unprocessed red meat and processed meat using data from the National Health and Nutrition Examination Survey (2015–2016 and 2017–2018). Items containing red or processed meat were classified into thirteen mutually exclusive food groups. For highly consumed food groups (≥10 % of meat intake), contribution to meat intake was further assessed by source, sex, income and education.

**Setting:**

Nationally representative sample of the US population.

**Participants:**

Teens (aged 12–19 years) and adults (aged ≥20 years) who reported meat consumption (*n* 8178).

**Results:**

Meat mixed dishes (18·6 % (95 % CI 16·2, 20·9)), burgers (17·3 % (95 % CI 15·3, 19·3)) and beef excluding ground (17·0 % (95 % CI 13·8, 20·1)) were the top contributors to unprocessed red meat intake. For processed meat, four food groups made up about four-fifths of total intake: cold cuts and cured meats (37·7 % (95 % CI 34·6, 40·8)), sausages and frankfurters (20·3 % (95 % CI 18·6, 22·0)), bacon (14·0 % (95 % CI 12·3, 15·6)) and pizza (10·1 % (95 % CI 8·7, 11·5)). Fast-food restaurants were the top source for burgers and pizza, whereas stores were the top source for all other highly consumed food groups. Few differences were seen in patterns of intake by sociodemographic characteristics.

**Conclusions:**

No single food group accounts for a majority of meat intake in the USA. Many behaviour change opportunities for healthier, more sustainable substitutions exist.

The 2020 US Dietary Guidelines Advisory Committee report concluded that there is ‘strong’ evidence that dietary patterns characterised by a higher intake of red or processed meat are associated with an increased risk of all-cause mortality and CVD, and ‘moderate’ evidence of increased risk of overweight and obesity, type 2 diabetes and colorectal cancer^(1)^. Unprocessed red meat is a good source of protein and micronutrients such as Fe and vitamin B_12_^(2)^. However, in high-income contexts such as the USA, most of the population (92 %) has adequate intakes of vitamin B_12_^(3)^, intake of protein far surpasses minimum requirements^(4)^, and the prevalence of Fe deficiency anaemia – the most common nutrient deficiency in the USA – is 10·4 % among females and 5·2 % among males^(5)^, much lower than that seen in lower-income contexts. At the same time, meta-analyses suggest that high intake of unprocessed meat is harmful for health^(6–8)^, associated with a 16 % higher risk of CHD^(9)^ and 19 % higher risk of type 2 diabetes^(10)^. The harmful effects of high intakes of processed meat are even greater^(6–8)^.

In addition to the adverse health effects associated with red and processed meat consumption, there are important environmental consequences of intensive meat production, with beef production being particularly harmful^(11)^. Specifically, US beef production requires twenty-eight times more land, eleven times more irrigation water and results in five times more greenhouse gases, compared to the average of other livestock categories (dairy, poultry, pork and eggs)^(12)^. Meats (beef, pork, chicken and other meats) account for 57 % of total greenhouse gas emissions in US diets, with 81 % of those meat-related emissions coming from beef alone^(13)^. In order to optimise both human nutrition and planetary health, the EAT-*Lancet* commission recommends consuming no more than 98 g/week of red meat and especially low intakes of processed meat^(14)^. Similarly, the American Heart Association 2020 Strategic Impact Goals for diet include reducing processed meat to none or ≤2 servings/week or about 100 g/week^(15)^. However, mean intake in the USA is much higher than these targets, at 284 g/week for unprocessed red meat and 187 g/week for processed meat^(16)^.

Few previous studies have evaluated which food groups contribute to unprocessed red meat and processed meat intake in the USA. An analysis of national dietary intake data from 1999–2016 found that lunch meats, sausages, hot dogs and ham account for nearly 90 % of processed meat intake but did not evaluate food groups contributing to unprocessed red meat intake^(16)^. Another recent analysis used the What We Eat in America (WWEIA) food categories to evaluate the American diet with respect to the Global Burden of Disease recommendations but did not disaggregate dietary sources of red and processed meat^(17)^. All of these studies are outdated – none used the latest round (2017–2018) of national dietary intake data.

In addition, few studies have examined the association of sociodemographic characteristics with meat intake^(16,18,19)^. The latest study to explore these associations found that income is not significantly related to unprocessed red meat or processed meat intake, but that unprocessed red meat intake is higher in individuals aged 35 to 64 years (compared to younger and older individuals) and in individuals with a high school education or less^(16)^. These differences are important given that dietary choices are influenced by income and educational background, among other factors^(20)^. Indeed, in a national survey, cost was identified as the most important reason for reducing meat intake among lower-income individuals^(21)^. Yet, how food groups contributing to meat intake differ according to sociodemographic characteristics has not been explored.

In order to identify opportunities to intervene on consumer behaviour, it is important to understand the way in which meat is prepared and consumed. Americans frequently eat meat in mixed dishes such as tacos, pizza or pasta, but the meat eaten in these food groups may be overlooked relative to foods such as steak or pork chops that only contain red meat^(17)^. The WWEIA Food Categories, which classify all foods reported in the National Health and Nutrition Examination Survey (NHANES) into mutually exclusive food groups based on usage and nutrition^(22,23)^, can be leveraged to better understand not only how much meat Americans are eating, but also the myriad ways in which meat is consumed. Previous studies have evaluated the contribution of different food groups to specific nutrients such as protein^(19)^, saturated fat^(24,25)^ and Na^(25–27)^. None has comprehensively quantified the contribution of different food groups to red and processed meat intake.

The primary objective of this study was to identify the food groups that contribute the most to red and processed meat intake in American diets using the most recently available nationally representative data. Secondary objectives included evaluating differences in the contribution of food groups to red and processed meat intake by (1) sociodemographic characteristics and (2) source (i.e. where meat is purchased). Results will inform the development of strategies to reduce red and processed meat consumption.

## Methods

### Data sources

This study used data from a single-day dietary recall in the 2015–2016 and 2017–2018 waves of NHANES. NHANES is a repeated cross-sectional survey that uses multistage probability design to sample the civilian, non-institutionalised population residing in all fifty states and the District of Columbia^(28)^. Trained interviewers used the US Department of Agriculture (USDA) Automated Multiple Pass Method to collect data on individual food intake^(29)^. Participants were asked to recall all foods and beverages they consumed the previous day^(29)^. Measuring guides were used to assist with approximating the portion sizes of consumed foods^(29)^.

Eligible participants for this analysis were teens (12–19 years)^(30)^ and adults (20 years and older)^(31)^ who had 1 d of valid dietary intake data and were neither pregnant nor lactating.

### Derivation of food groups and contributions to meat intake

NHANES 24-h dietary recall data were merged to the USDA Food Patterns Equivalent Database (FPED) to obtain grams of meat consumed per d. FPED is a tool that compliments each cycle of the NHANES survey and uses standard recipes to disaggregate mixed dishes into their component ingredients, such that the weight of cooked meat was disaggregated from the weight of the remaining dish ingredients^(32,33)^. Using FPED, unprocessed red meat intake was defined as follows: (1) FPED ‘Meat’ component, including mammalian muscle meat from beef, veal, pork, lamb and/or game meat and (2) FPED ‘Organ Meat’ component, including organ meat from beef, veal, pork, lamb, game and/or poultry^(32)^. Processed meat intake was defined using the FPED ‘Cured Meat’ component, which includes bacon, frankfurters, hot dogs, sausages, pepperoni, jerky and luncheon meats that are made from beef, pork or poultry^(32)^. Total red and processed meat intake was the total of unprocessed red meat intake + processed meat intake, that is, the total of FPED Meat, FPED Organ Meat and FPED Cured Meat. For dishes in which there were both unprocessed red meat and processed meat components, the FPED classification scheme enabled the disaggregation of the weight of unprocessed red meat and processed meat so that they could be analysed separately.

Ounce equivalent weights were converted from FPED to grams using a conversion factor of 1 ounce = 28·35 g^(17)^. Individuals with zero reported intake of both unprocessed red and processed meat were retained in the analytic dataset for survey-weighting purposes but excluded from further analyses (see online Supplemental Fig. 1).

The WWEIA Food Categories are released as a tool with each cycle of NHANES and classify all NHANES into 167 mutually exclusive categories by grouping similar foods together based on usage and nutrient content^(22,23)^. NHANES data, with grams of red and processed meat derived from FPED, were merged to WWEIA by food code. Thirteen food groups were derived from the twenty-eight WWEIA Food Categories containing red or processed meat (Table 1)^(22)^. WWEIA categories that contributed less than 1 % of total meat intake by weight were collapsed into ‘Other dishes’.

**Table 1:** Contributions of 28 What We Eat in America (WWEIA) food groups to analytic categories, NHANES 2015–2018

Analytic food group	WWEIA food group	Contribution to total meat intake by weight, %*	Example foods
Cold cuts and cured meats	Cold cuts and cured meats	15·9 %	‘Bologna, NFS’; ‘Ham, prepackaged or deli, luncheon meat’; ‘Beef jerky’; ‘Corned beef, canned, ready-to-eat’; ‘Salami, made from any type of meat, reduced fat’
Burgers	Burgers	11·0 %	‘Hamburger, 1 medium patty, with condiments, on wheat bun’; ‘Double cheeseburger, 2 medium patties, plain, on bun, from fast food/restaurant’
Beef-excludes ground	Beef-excludes ground	10·2 %	‘Beefsteak, fried, lean and fat eaten’; ‘Beefsteak, breaded or floured, baked or fried, lean only eaten’; ‘Pork steak or cutlet, broiled or baked, lean only eaten’
Meat mixed dishes	Meat mixed dishes	9·3 %	‘Meatloaf made with beef, with tomato-based sauce’; ‘Shepherd’s pie with beef’; ‘Pork, potatoes, and vegetables including carrots, broccoli, and/or dark-green leafy; gravy’; ‘Cornmeal mush, green cabbage, beef with tomato sauce’
Sausages and frankfurters	Sausages	5·4 %	‘Chorizo’; ‘Pork sausage, reduced fat’; ‘Polish sausage’; ‘Beef sausage’; ‘Bratwurst’; ‘Sausage, NFS’
	Frankfurter sandwiches	3·2 %	‘Frankfurter or hot dog sandwich, beef, plain, on white bun’; ‘Corn dog, frankfurter or hot dog with cornbread coating’; ‘Pig in a blanket, frankfurter or hot dog wrapped in dough’
	Frankfurters	0·7 %	‘Frankfurter, wiener, or hot dog, NFS’; ‘Frankfurter or hot dog, meat and poultry’; ‘Frankfurter or hot dog, beef and pork’; ‘Frankfurter or hot dog, beef’
Mexican mixed dishes	Mixed dishes – Mexican	7·9 %	‘Burrito with meat, beans, rice, and sour cream’; ‘Empanada, Mexican turnover, filled with meat and vegetables’; ‘Gordita, sope, or chalupa with meat’; ‘Soft taco with meat and sour cream, from fast food’
Pork	Pork	7·2 %	‘Pork chop, fried, lean only eaten’; ‘Pork, spareribs, cooked, lean and fat eaten’; ‘Pork roast, loin, cooked, lean and fat eaten’
Grain-based mixed dishes	Mixed dishes – grain-based	5·4 %	‘Jambalaya with meat and rice’; ‘Pasta, whole grain, with tomato-based sauce, ready-to-heat’; ‘Macaroni or noodles with cheese and meat, prepared from Hamburger Helper mix’; ‘Turnover, meat- and cheese-filled, no gravy’
Bacon	Bacon	4·9 %	‘Pork bacon, NS as to fresh, smoked or cured, cooked’; ‘Bacon, NS as to type of meat, cooked’
Pizza	Pizza	4·8 %	‘Pizza with extra meat, medium crust’; ‘Pizza with meat and vegetables, from frozen, thin crust’; ‘Calzone, with meat and cheese’; ‘Pizza rolls’
Eggs and egg sandwiches	Eggs and omelettes	1·5 %	‘Egg, Benedict’; ‘Egg omelette or scrambled egg, with cheese and meat, NS as to fat added in cooking’; ‘Egg omelette or scrambled egg, with meat and vegetables other than dark-green and/or tomatoes, fat added in cooking’
	Egg/breakfast sandwiches	2·3 %	‘Egg, cheese, and ham on English muffin’; ‘Egg, cheese, and ham on English muffin’; ‘Burrito, taco, or quesadilla with egg and breakfast meat, from fast food’
Other sandwiches	Other sandwiches	1·2 %	‘Meatball and spaghetti sauce submarine sandwich’; Roast beef sandwich with cheese’; ‘Gyro sandwich (pita bread, beef, lamb, onion, condiments), with tomato and spread’
	Chicken/Turkey sandwiches	0·3 %	‘Chicken, bacon, and tomato club sandwich, with lettuce and spread’; ‘Turkey and bacon submarine sandwich, with cheese, lettuce, tomato and spread’
Other	Soup	2·9 %	‘Beef noodle soup, home recipe’; ‘Dark-green leafy vegetable soup with meat, Asian style’; ‘Beef vegetable soup, home recipe, Mexican style’; ‘Pho’; ‘Pepperpot soup’; ‘Bean soup, NFS’
	Mixed dishes – Asian	1·9 %	‘Beef chow mein or chop suey with noodles’; ‘Rice, fried, with pork’; ‘Sweet and sour pork’; ‘Egg roll, with beef and/or pork’; ‘Dumpling, steamed, filled with meat, poultry, or seafood’
	Lamb, goat and game	0·8 %	‘Lamb, loin chop, cooked, lean only eaten’; ‘Goat, boiled’; ‘Venison/deer steak, cooked, NS as to cooking method’
	Liver and organ meats	0·3 %	‘Gizzard, cooked’; ‘Hog maws, cooked’; ‘Beef liver, fried’
	Cheese	<0·1 %	‘Cheese with nuts’
	Mixed dishes – Bean/vegetable-based†	<0·1 %	‘Beans and rice, with meat’; ‘Stuffed pepper, with meat’; ‘Mushrooms, stuffed’
	Poultry mixed dishes	<0·1 %	‘Adobo, with noodles’; ‘Chicken or turkey, breaded, fried, garden salad with bacon and cheese, chicken and/or turkey, bacon, cheese, lettuce and/or greens, tomato and/or carrots, other vegetables, no dressing’
	Plant-based protein foods – beans, peas, legumes	<0·1 %	‘Pork and beans’; ‘Beans, dry, cooked with ground beef’; ‘Stewed chickpeas, with potatoes, Puerto Rican style’
	Pretzels/snack mix†	<0·1 %	‘Pretzels, soft, ready-to-eat, topped with meat’
	Seafood mixed dishes	<0·1 %	‘Gumbo with rice’; ‘Gumbo, no rice’
	White potatoes	<0·1 %	‘Potato, baked, peel not eaten, with meat’; ‘Potato, french fries, with chilli and cheese, fast food/restaurant’; ‘Potato skins, with cheese and bacon’; ‘Potato, scalloped, from fresh, with meat’

* Values are unweighted contribution to mean intake of total meat among consumers.

† From 2017–2018 NHANES only.

### Source and sociodemographic characteristics

The source of the food group was based on the answer to the question, ‘Where did you get this or most of the ingredients for this?’^(29)^. Source was classified into four categories: stores (grocery stores, supermarkets, convenience stores and stores – no additional info); fast-food restaurants; full-service restaurants and others (including cafeterias, dining halls, community meal programmes, gifts and others)^(34)^.

For age, participants were classified as teens (aged 12–19 years)^(30)^ or adults (aged 20 years and older)^(31)^. Teens were included in this analysis to capture a period of development during which individuals are heavily targeted by marketing, susceptible to peer influence, and form their food preferences^(35,36)^. For income, the Poverty Income Ratio (PIR), a measure of family income relative to the Federal Poverty Level, was used to create income categories. Family income was categorised as low (PIR 0–185 %), middle (PIR 186–400 %) and high (PIR >400 %)^(37)^. For educational attainment, adult participants were classified as low (less than high school degree), middle (high school graduate/GED or equivalent or some college/associate degree) and high (college graduate or above)^(28,31)^. Educational attainment of the household reference person was used to classify teens into low (less than high school degree), middle (high school/GED or equivalent or some college/associate degree) and high (college graduate or above) in accordance with the NHANES Sample Design and Estimation Procedures Manual^(28)^ and specifications in the NHANES Data Documentation Codebook^(31)^.

### Statistical analyses

Descriptive statistics were used to quantify the per cent contribution (95 % CI) of each food group to total red and processed meat, unprocessed red meat, and processed meat intake. Results were obtained overall and, for food groups that accounted for ≥10 % of either unprocessed red or processed meat intake, further by source, age, sex, income and educational attainment. The relative CI widths of the proportional measures were examined to assess the reliability of estimates in accordance with recommendations of the National Center of Health Statistics^(38)^. Adjusted Wald tests were used to test for significant differences between groups. Mean meat intakes (95 % CI) were calculated for descriptive purposes. Due to differences in energy needs between teens and adults, mean daily intakes are presented separately for these two groups. All analyses were conducted in Stata SE 16^(39)^, using Stata’s svy and subpop commands with the NHANES survey weights to account for complex survey design.

## Results

Of the 11 808 eligible individuals aged 12 years or older, not pregnant or lactating, with dietary intake data, 8178 (survey-weighted proportion: 71·0 % (95 % CI 69·4, 72·7)) reported eating meat in the preceding 24 h (see online Supplemental Fig. 1). The final analytic sample thus included 8178 eligible consumers, about half of whom (52·9 %) reported eating meat once in the preceding 24 h, while the remaining 47·1 % reported eating meat two or more times (Table 2). The mean intake of total red and processed meat was 104·9 (95 % CI 101·7, 108·0) g/d; for adults, mean intake of unprocessed red meat was 65·6 (95 % CI 62·3, 68·8) g/d, and mean intake of processed meat was 38·9 (95 % CI 36·5, 41·3) g/d (Table 2). For teens, mean intake was 93·5 (95 % CI 86·7, 100·4) g/d for total red and processed meat, 56·1 (95 % CI 49·6, 62·5) g/d for unprocessed red meat and 37·4 (95 % CI 33·3, 41·5) g/d for processed meat (Table 2). Stores were the most common source for meat-containing dishes, contributing 58·3 % of meat (Table 2). Fast-food restaurants were also an important source for meat, contributing nearly 20 % of meat for adults and more than 25 % of meat for teens (Table 2).

**Table 2:** Characteristics of participants aged 12 years and older that reported meat consumption on a single-day dietary recall, NHANES 2015–2018

	Total *n* 8178	%	Adults aged ≥20 years *n* 6728	%	Teens aged 12–19 years *n* 1450	%
Sex*						
Male	53·1	4336	52·7	3538	55·8	798
Female	47·9	3870	47·3	3190	44·2	652
Age*						
12–19	9·9	622	–		100·0	1450
20–29	17·8	576	17·7	997	–	
30–39	15·8	515	17·2	1077	–	
40–49	13·7	495	16·0	1064	–	
50–59	16·3	542	19·1	1123	–	
60–69	13·9	692	16·0	1302	–	
70–79	8·9	370	9·4	726	–	
80+	3·7	218	4·7	439	–	
Educational attainment*,†						
Low	12·0	1595	11·1	1277	19·2	318
Medium	59·7	4757	58·6	3941	58·3	816
High	28·3	1746	29·0	1504	22·5	242
Income*,‡						
Low	26·1	3027	24·5	2367	37·7	660
Medium	30·4	2500	30·4	2084	30·1	416
High	35·2	1836	36·6	1597	24·2	239
Missing	8·4	815	8·4	680	8·0	135
Purchase location of total red and processed meat§						
Stores‖	58·3	7529	59·6	6515	51·1	1014
Fast-food restaurants	20·5	2645	19·6	2140	25·4	505
Full-service restaurants	10·6	1369	11·3	1238	6·6	131
Other¶	10·7	1376	9·5	1041	6·9	335
Number of meat-eating occasions in a single day*						
1	52·9	4385	52·2	3600	58·4	785
2	33·2	2633	33·9	32·7	29·8	411
3 or more	13·9	1160	14·0	978	13·2	182
Mean intake (grams)**						
Unprocessed red meat						
Mean	64·5		65·6		56·1	
95 % CI	61·4, 67·6		62·3, 68·8		49·6, 62·5	
Processed meat						
Mean	38·8		38·9		37·4	
95 % CI	36·4, 41·1		36·5, 41·3		33·3, 41·5	
Total meat						
Mean	103·5		104·9		93·5	
95 % CI	100·5, 106·5		101·7, 108·0		86·7, 100·4	

* Values are weighted % (unweighted *n*). Weighted % accounts for complex survey weights.

† Family income was categorised as low (Poverty Income Ratio (PIR) 0–185 %), middle (PIR 186–400 %) and high (PIR >400 %).

‡ Education was defined as low (less than high school), medium (high school graduate/GED or equivalent or some college/associate degree) and high (college graduate or above). Education categories for teens were defined using the household reference person’s educational attainment.

§ Values are unweighted % (unweighted *n*) of all reported meat-eating occasions.

‖ Stores include grocery stores, supermarkets, convenience stores and stores – no additional info.

¶ Includes cafeterias, dining halls, community meal programmes, gifts and other.

** Values are mean weight in grams (95 % CI) consumed among consumers only and account for complex survey weights.

The contributions of different food groups to intake are shown in Table 3 and Supplemental Fig. 2. Cold cuts and cured meats accounted for 18·1 % (95 % CI 16·2, 20·0) of total red and processed meat, while burgers accounted for 11·0 % (95 % CI 9·9, 12·0), beef-excludes ground accounted for 10·3 % (95 % CI 8·8, 11·8) and meat mixed dishes accounted for 10·2 % (95 % CI 9·3, 11·0) (Table 3; see online Supplemental Fig. 2). Meat mixed dishes were the top contributor to unprocessed red meat intake, accounting for 18·6 % (95 % CI 16·2, 20·9) of total unprocessed red meat intake, followed by burgers (17·3 % (95 % CI 15·3, 19·3)), beef-excludes ground (17·0 % (95 % CI 13·8, 20·1)) and Mexican mixed dishes (14·2 % (95 % CI 10·4, 18·1)) (Table 3; see online Supplemental Fig. 2). Pork accounted for 11·8 % (95 % CI 9·4, 14·3) of unprocessed red meat consumption (Table 3; see online Supplemental Fig. 2). For processed meat, cold cuts and cured meats made up more than a third (37·7 % (95 % CI 34·6, 40·8)) of total intake (Table 3; see online Supplemental Fig. 2). Sausages and frankfurters, bacon and pizza were also important contributors, accounting for 20·3 % (95 % CI 18·6, 22·0), 14·0 % (95 % CI 12·3, 15·6) and 10·1 % (95 % CI 8·7, 11·5) of processed meat intake, respectively (Table 3; see online Supplemental Fig. 2).

**Table 3:** Contribution of thirteen food groups to total red and processed meat, unprocessed red meat and processed meat intake in US diets, NHANES 2015–2018*

	Total red and processed meat		Unprocessed red meat		Processed meat	
	Weighted mean %	95 % CI	Weighted mean %	95 % CI	Weighted mean %	95 % CI
Cold cuts and cured meats	18·1	16·2, 20·0	–		37·7	34·6, 40·8
Burgers	11·0	9·9, 12·0	17·3	15·3, 19·3	–	
Beef-excludes ground	10·3	8·8, 11·8	17·0	13·8, 20·1	–	
Meat mixed dishes	10·2	9·3, 11·0	18·6	16·2, 20·9	1·6	1·1, 2·1
Sausages and frankfurters	9·6	8·7, 10·4	–		20·3	18·6, 22·0
Mexican mixed dishes	7·1	5·9, 8·3	14·2	10·4, 18·1	–	
Pork	6·3	5·3, 7·2	11·8	9·4, 14·3	–	
Grain-based mixed dishes	5·6	4·9, 6·4	7·9	6·0, 9·8	1·7	1·2, 2·3
Bacon	5·2	4·3, 6·0	–		14·0	12·3, 15·6
Pizza	4·7	4·1, 5·3	1·3	0·2, 2·4	10·1	8·7, 11·5
Eggs and egg sandwiches	3·3	2·8, 3·8	<0·1	0·0, 0·1	8·1	7·0, 9·2
Other sandwiches	2·0	1·6, 2·3	3·0	2·3, 3·8	2·6	1·9, 3·3
Other	6·7	6·1, 7·3	8·4	7·2, 9·6	3·8	2·9, 4·7

* Values are survey-weighted mean percent (95 % CI) of total grams consumed.

Differences were observed in patterns of meat intake for adults and teens for several key food groups (see online Supplemental Table 1). Meat mixed dishes accounted for a larger proportion of adults’ total red and processed meat intake (10·7 % (95 % CI 9·7, 11·6) of intake for adults *v*. 6·3 % (95 % CI 4·5, 8·0) for teens, *P* < 0·001) (see online Supplemental Table 1). Pork accounted for 6·6 % (95 % CI 5·5, 7·6) of total red and processed meat intake for adults, compared to 4·0 % (95 % CI 2·7, 5·3, *P* < 0·01) for teens (see online Supplemental Table 1). Conversely, a smaller proportion of adults’ total meat intake came from burgers (10·5 % (95 % CI 9·4, 11·5) for adults *v*. 14·9 % (95 % CI 12·3, 17·5) for teens, *P* < 0·01) and pizza (4·2 % (95 % CI 3·6, 4·9) for adults *v*. 9·0 % (95 % CI 6·7, 11·2) for teens, *P* < 0·001). When restricted to unprocessed red meat only, the differences for the contribution of meat mixed dishes (17·6 % (95 % CI 15·7, 19·5) for adults *v*. 11·6 % (95 % CI 8·6, 14·6) for teens, *P* < 0·01) persisted (see online Supplemental Table 1). Additionally, for unprocessed red meat, a smaller proportion of adults’ intake came from burgers (18·5 % (95 % CI 16·7, 20·4)) as compared to that of teens (28·5 % (95 % CI 24·3, 32·8), *P* < 0·001) (see online Supplemental Table 1). Finally, a smaller proportion of adults’ processed meat intake came from pizza ((9·1 % (95 % CI 7·8, 10·3) for adults *v*. 17·6 % (95 % CI 13·7, 21·4) for teens, *P* < 0·001) (see online Supplemental Table 1).

Stores were the most common source for cold cuts and cured meat, beef-excludes ground, sausages and frankfurters, meat mixed dishes, grain-based mixed dishes, Mexican mixed dishes, pork and bacon (Table 4). Fast-food restaurants were the top source for burgers and pizza (Table 4). While full-service restaurants were not the top-cited source for any food group, they accounted for 22·2 % (95 % CI 18·3, 26·2) of beef-excludes ground intake (Table 4).

**Table 4:** Contributions of thirteen food groups to total red and processed meat intake in US diets, according to source, NHANES 2015–2018

	Stores*		Fast-food restaurants		Full-service restaurants		Other†	
	Weighted %	95 % CI	Weighted %	95 % CI	Weighted %	95 % CI	Weighted %	95 % CI
Cold cuts and cured meats	81·2	78·0, 84·5	5·4	3·8, 6·9	5·2	3·3, 7·0	8·1	5·8, 10·5
Burgers	30·1	24·7, 35·5	50·6	44·8, 56·3	11·0	6·6, 15·4	8·3	6·1, 10·6
Beef-excludes ground	60·2	55·8, 64·6	8·5	6·3, 10·7	22·2	18·3, 26·2	8·9	6·4, 11·4
Meat mixed dishes	72·5	68·4, 76·6	5·6	4·0, 7·1	9·6	6·6, 12·6	11·7	9·0, 14·4
Sausages and frankfurters	68·1	64·1, 72·0	10·3	6·8, 13·9	5·8	3·4, 8·2	14·7	11·7, 17·7
Mexican mixed dishes	41·5	34·7, 48·2	31·7	23·8, 39·6	18·6	14·0, 23·2	8·2	5·6, 10·8
Pork	73·4	67·4, 79·4	7·0	4·9, 9·0	10·7	7·1, 14·4	8·7	5·1, 12·3
Grain-based mixed dishes	78·2	74·4, 82·0	4·4	3·0, 5·9	6·4	4·4, 8·4	9·9	6·2, 13·5
Bacon	57·5	52·4, 62·7	15·2	11·2, 19·3	18·2	14·0, 22·4	8·9	6·6, 11·3
Pizza	28·6	24·6, 32·7	52·2	47·5, 56·9	13·8	9·0, 18·6	5·3	3·4, 7·3

* Stores include grocery stores, supermarkets, convenience stores and stores – no additional info.

† Other sources include cafeterias, dining halls, sporting events, gifts and community meal programmes.

Bacon accounted for 6·5 % (95 % CI 5·2, 7·8) of female total red and processed meat intake, which was significantly higher than its contribution among males (4·0 % (95 % CI 3·0, 5·0), *P* < 0·01, see online Supplemental Table 2). Pork accounted for a smaller proportion of total red and processed meat intake for those in the high-income category compared to those in the low-income category (4·1 % (95 % CI 3·0, 5·3) for high-income *v*. 7·6 % (95 % CI 6·2, 9·0) for low-income, *P* < 0·001, see online Supplemental Table 2). The contribution of Mexican mixed dishes was smaller for individuals with a college degree or above (5·2 % (95 % CI 3·6, 6·7)) than for those with less than high school education (11·0 % (95 % CI 7·8, 14·3), *P* < 0·001, see online Supplemental Table 2).

## Discussion

This study provides the most up-to-date information on how Americans are consuming red and processed meat. Overall, and consistent with prior literature, average meat intake among consumers – 105 g/d for adults and 94 g/d for teens – exceeds recommendations outlined by the EAT-*Lancet* Commission on Food, Planet, Health and the American Heart Association that cite a target of about 100 g of total meat intake per week^(40,41)^. Three of the thirteen food groups evaluated – burgers, beef-excludes ground and meat mixed dishes – accounted for 17–19 % of unprocessed red meat intake each. Mexican mixed dishes were also significant contributors, accounting for 14 % of unprocessed red meat intake. For processed meat, four food groups made up about four-fifths of total intake: cold cuts and cured meats, sausages and frankfurters, bacon, and pizza. Cold cuts and cured meats alone accounted for over a third of all processed meat intake in the US diet, almost double the contribution of sausages and frankfurters, the second-highest contributor. Nonetheless, no one food group stood out as being the main contributor to red meat intake, and so interventions could target a variety of food groups, such as sandwich meats, burgers, sausages, traditional US meat mixed dishes such as meatloaf. Replacing even one of these food groups with healthier, more sustainable foods could have a meaningful impact on total meat intake in the USA. Changing dietary behaviour is challenging, particularly for processed meat, which has remained unchanged over the past 18 years, and unprocessed red meat, which has declined very little over the same period^(16)^. However, a recent systematic review found that several randomised controlled trials showed promising effects on meat reduction, especially reducing meat portion sizes and providing meat alternatives^(42)^. Moreover, an estimated 16 % of Americans were considered ‘potential changers’ should the Dietary Guidelines for Americans include information and suggestions for sustainable diets^(43)^ and 55 % of Americans reported reducing the amount of processed meat they consumed in the past 3 years and 41 % reduced red meat^(21)^. This previous literature supports the possibility of future declines in meat intake, particularly with targeted, evidence-based interventions.

Teens as compared to adults ate less total red and processed meat and were more likely than adults to get their meat from burgers and pizza, which were primarily sourced from fast-food restaurants and accounted for 28·5 % of teens’ unprocessed red meat intake and 17·6 % of their processed meat intake, respectively. Previous studies have shown that teens are more motivated to adopt plant-based diets for ethical reasons including environmental concern as compared to older adults who are more motivated to adopt similar diets for health reasons^(44)^. Emphasising the environmental benefits of meat reduction or choosing plant-based alternatives may be especially impactful for this demographic group, particularly because they already eat less meat relative to American adults in this study. The increasing availability of biomimicry burgers in the USA (e.g. the Beyond Burger and Impossible Burger), including at major fast-food chains^(45,46)^, represents one promising opportunity for replacement of red meat (in particular, beef) with more sustainable plant-based burgers. Additionally, pizza is an emerging market for biomimicry products, as supported, for example, by a national pizza chain launching a Beyond Pepperoni pizza in 2021^(47,48)^. Additionally, many popular pizzas are already meat-free, providing an opportunity to shift behaviour away from meat towards toppings such as vegetables, mushrooms or plain cheese^(49)^.

With regard to processed meat, cold cuts and cured meats made up more than one-third of intake. Sausages and frankfurters made up another 20 %, while bacon and pizza accounted for about 14 % and 10 %, respectively. These findings are consistent with a recent analysis of NHANES data from 2015 to 2016 that reported the following gram-weight proportional contributions to processed meat: 39·3 % from lunch meat, 24·4 % from sausage, 9·4 % from hot dogs, 9·4 % from ham and 4·6 % from bacon^(16)^. It is clear from these analyses that cold cuts and cured meats are a major contributor to processed meat intake in the USA and may be consumed as part of a broader unhealthy dietary pattern^(50)^. Thus, they should be a focus for food policy, including school-based interventions.

Considering health and environmental impacts, it is important not to treat all meat as homogenous. For example, processed meat has stronger adverse effects on health than unprocessed meat^(51,52)^, and beef has a much higher carbon footprint than pork^(53)^. In this study, burgers and beef-excludes ground accounted for about 35 % of all unprocessed red meat consumption by Americans, whereas cold cuts and cured meats – which have relatively more pork than beef products – accounted for most processed meat consumption. Future research should explore how public health interventions or new products targeting specific food groups – for example, plant-based burgers, sausages and pepperoni – might have differential environmental impacts and identify which interventions and products optimise both health and environmental outcomes.

There are several strengths and limitations of this study. We used data from the two most recent waves of NHANES and applied weights such that estimates are nationally representative. These are best practices for obtaining reliable estimates of population-level red and processed meat intake^(54)^. However, no food grouping system is perfect. This meant that some foods in the WWEIA Food Categories – and therefore the derived analytic food groups – could have fallen under multiple classifications. For example, ‘Meat and hominy soup, Mexican style’ was classified as a ‘Soup’ but could have reasonably been classified as ‘Mexican mixed dish’. Additionally, the FPED food groups do not allow for analysing beef separately from pork, and poultry is included in processed foods and organ meats. Given that the environmental impact of beef, pork and poultry varies substantially – for example, beef has approximately eight times the carbon footprint of chicken (32·8 CO_2_ eq/kg *v*. 4·2 CO_2_ eq/kg per kg of edible boneless weight)^(13)^ – our ability to conclusively comment on the environmental impact of observed changes is limited. While we may have missed some combination foods, using the WWEIA Food Categories facilitates comparisons to previous studies. Related to these two limitations, the 2020 US Dietary Guidelines Advisory Committee report highlighted the need for future work on eating patterns to more clearly differentiate lean meat, red meat and processed meat^(1)^. Findings of this study may be used to refine these definitions and also to improve translation into practice.

This study of how Americans eat red and processed meat indicates that burgers, beef, mixed dishes, cold cuts, sausages, frankfurters, pizza and bacon contributed more than 10 % to either unprocessed red or processed meat intake and are major routes of exposure to meat. Further efforts are needed to promote reduction in top contributors such as cured meats, cold cuts, beef and pizza, either through the development of biomimicry products such as plant-based lunch meats and pepperoni, or other plant-based protein choices such as beans or nut-based spreads (e.g. peanut butter) that are already enjoyed by millions of Americans^(19,55)^. However, it will be important to closely monitor trends in protein choices to ensure that environmental gains are not traded at the cost of health (e.g. ensuring nut-based spreads are not high in added sugars). Additionally, it is important that culturally appropriate protein alternatives are available, accessible and affordable. Nonetheless, no one food group stood out as being the main contributor to red meat intake, and so interventions could target a variety of food groups, such as sandwich meats, burgers, sausages and traditional US meat mixed dishes such as meatloaf. Replacing even one of these food groups with healthier, more sustainable foods could have a meaningful impact on total meat intake in the USA in communities throughout the USA. Overall, the pattern of meat-eating in the USA is quite complex, and so a one-size-fits-all approach will not be sufficient for meeting meat reduction targets.

## Supplementary material

For supplementary material accompanying this paper visit https://doi.org/10.1017/S1368980022000416. click here to view supplementary material
