# Supplementary material for: How Americans eat red and processed meat: an analysis of the contribution of thirteen different food groups
Source: Public Health Nutr. 2022 Feb 21;25(5):1406–15. doi: 10.1017/S1368980022000416 (PMC9991741; doi:10.1017/S1368980022000416)
Supplement: Supplementary file 1 [file S1368980022000416sup001.docx]

**Supplemental Figure 1.** Flow chart of participant inclusion. Note that unprocessed red meat intake and processed meat intake sum to greater than total reporters due to participants who reported both red and processed meat intake and who were therefore included in both subgroups.


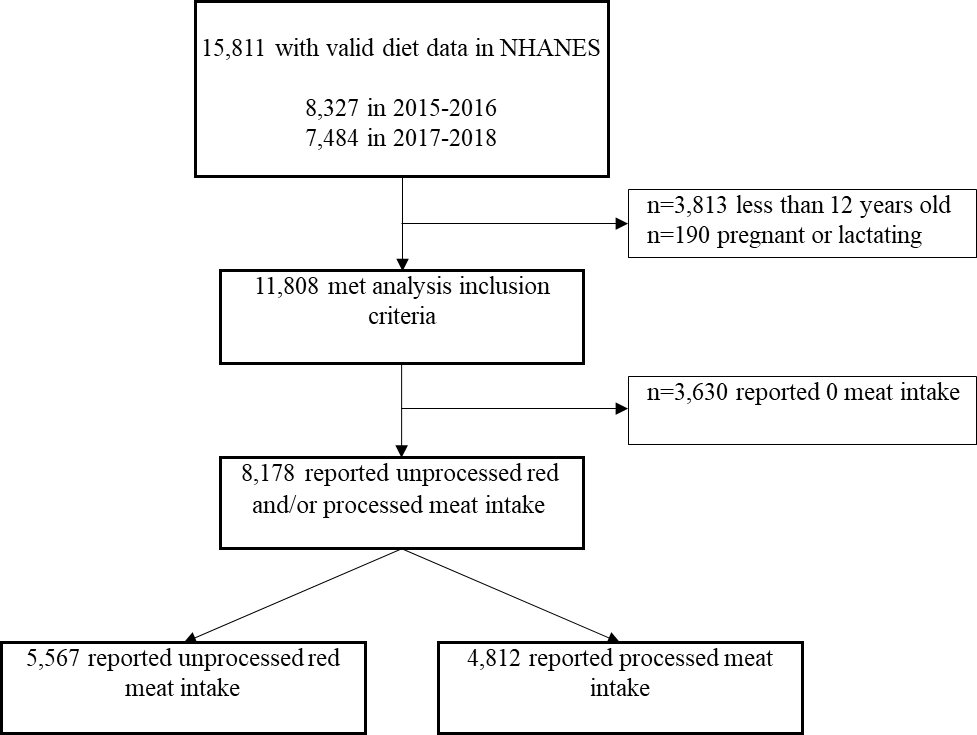


**Supplemental Figure 2.** Contribution of 13 food groups to total red and processed meat, unprocessed red meat, and processed meat intake in US diets, NHANES 2015-2018.^1^

^1^ Values are survey-weighted mean percent (95% confidence interval) of total grams consumed.

**
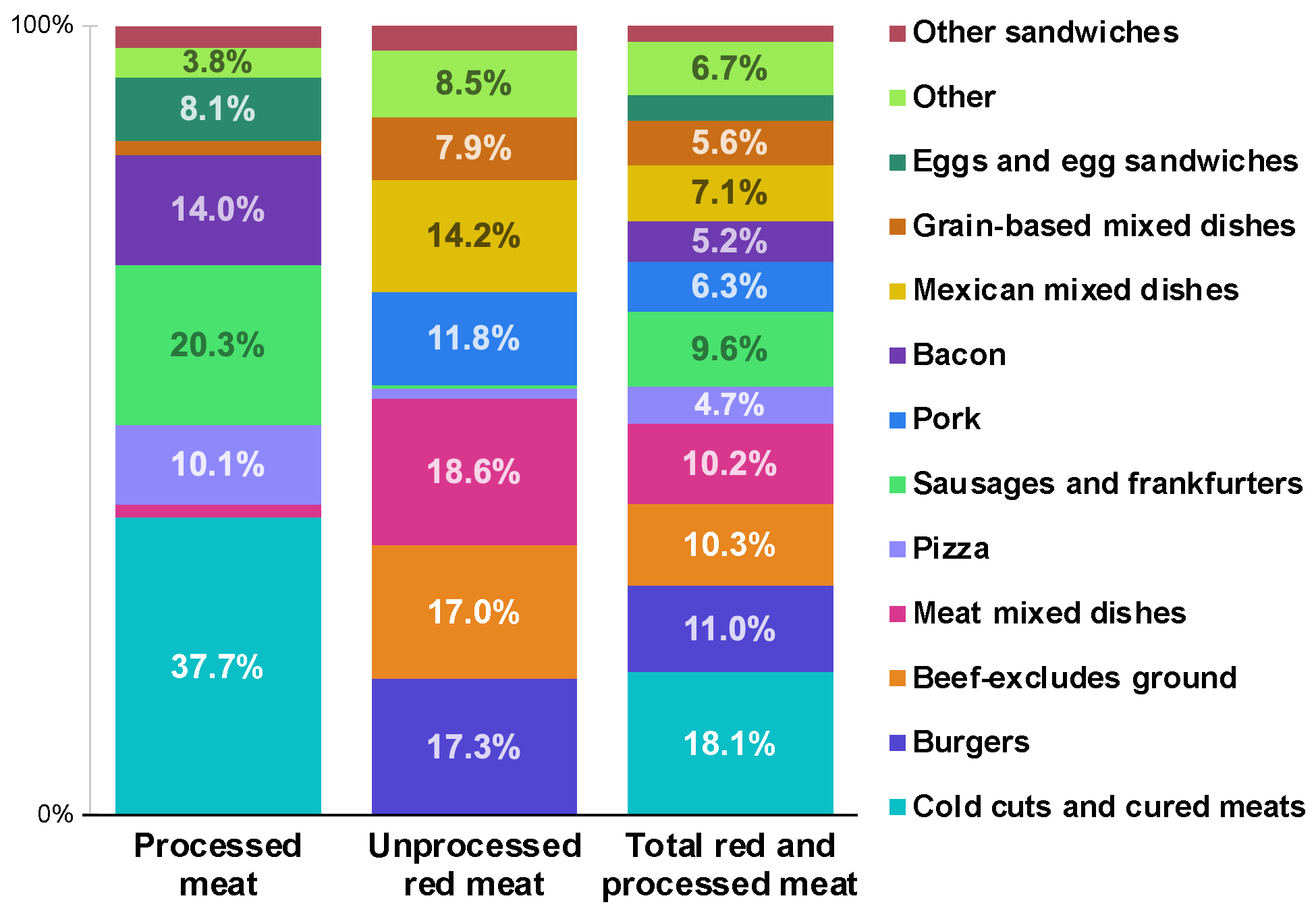
**

| **Supplemental Table 1.** Comparison of contribution of food groups to total red and processed meat, unprocessed red meat, and processed meat intake in adults and teens, NHANES 2015-2018.^1^ | | |
| --- | --- | --- |
|  | **Adults** | **Teens** |
| *Total red and processed meat* |  |  |
| Cold cuts and cured meats | 17.9 (16.0-19.8) | 19.9 (16.6-23.1) |
| Burgers | 10.5 (9.4-11.5) | 14.9 (12.3-17.5)** |
| Beef | 10.7 (9.1-12.3) | 7.4 (5.6-9.1)** |
| Meat mixed dishes | 10.7 (9.7-11.6) | 6.3 (4.5-8.0)*** |
| Sausages and frankfurters | 9.6 (8.6-10.6) | 9.3 (7.6-11.0) |
| Mexican mixed dishes | 6.8 (5.5-8.0) | 9.5 (6.9-12.1) |
| Pork | 6.6 (5.5-7.6) | 4.0 (2.7-5.3)** |
| Grain-based mixed dishes | 5.5 (4.8-6.3) | 6.2 (4.1-8.2) |
| Bacon | 5.2 (4.2-6.2) | 4.8 (3.9-5.7) |
| Pizza | 4.2 (3.6-4.9) | 9.0 (6.7-11.2)*** |
|  |  |  |
| *Unprocessed red meat* |  |  |
| Burgers | 18.5 (16.7-20.4) | 28.5 (24.3-32.8)*** |
| Beef | 17.7 (15.4-20.0) | 13.2 (10.2-16.3) |
| Meat mixed dishes | 17.6 (15.7-19.5) | 11.6 (8.6-14.6)** |
| Mexican mixed dishes | 11.9 (9.6-14.3) | 17.3 (13.1-21.6) |
| Pork | 11.0 (9.3-12.7) | 7.3 (4.9-9.7) |
| Grain-based mixed dishes | 8.7 (7.4-10.1) | 10.5 (6.7-14.2) |
|  |  |  |
| *Processed meat* |  |  |
| Cold cuts and cured meats | 37.6 (34.5-40.7) | 37.9 (32.3-43.5) |
| Sausages and frankfurters | 20.6 (18.7-22.4) | 18.7 (15.6-21.8) |
| Bacon | 14.3 (12.4-16.2) | 11.8 (9.9-13.7) |
| Pizza | 9.1 (7.8-10.3) | 17.6 (13.7-21.4)*** |
| ^1^ Values are survey-weighted mean percent (95% confidence interval) of total grams consumed.  *p<0.05, **p<0.01, ***p<0.001 for the difference in proportional contribution to meat intake. | | |

| **Supplemental Table 2**. Contributions of food groups to total red and processed meat, unprocessed red meat, and processed meat intake in US diets, according to sex, income, and educational attainment, NHANES 2015-2018.^1^ | | | | | | | | |
| --- | --- | --- | --- | --- | --- | --- | --- | --- |
|  | **Sex** | | **Income^2^** | | | **Educational Attainment^3^** | | |
|  | **Male** | **Female** | **Low** | **Middle** | **High** | **Low** | **Middle** | **High** |
| *Total red and processed meat* | | | | | | | | |
| Cold cuts and cured meats | 18.3 (16.3-20.2) | 17.9 (15.7-20.1) | 17.5 (15.1-19.9) | 18.9 (16.2-21.6) | 18.6 (16.3-20.9) | 15.2 (12.8-17.6) | 18.2 (15.9-20.6) | 19.0 (15.9-22.1) |
| Burgers | 12.0 (10.6-13.4) | 9.8 (8.5-11.1) | 12.1 (10.6-13.7) | 10.8 (8.8-12.9) | 10.7 (8.8-12.6) | 10.7 (8.8-12.6) | 12.3 (10.9-13.7) | 8.4 (6.3-10.5) |
| Beef - excludes ground | 11.4 (9.2-13.6) | 9.1 (7.6-10.6) | 8.7 (7.3-10.0) | 9.7 (7.9-11.5) | 11.7 (9.0-14.4) | 10.5 (8.4-12.5) | 9.8 (8.3-11.2) | 11.5 (8.0-14.9) |
| Meat mixed dishes | 9.0 (7.7-10.2) | 11.5 (10.4-12.7) | 9.2 (7.8-10.7) | 10.9 (9.0-12.8) | 10.4 (8.7-12.0) | 7.9 (6.4-9.4) | 10.2 (9.0-11.4) | 11.2 (9.1-13.2) |
| Sausages and frankfurters | 10.0 (8.7-11.2) | 9.1 (8.0-10.1) | 9.0 (7.8-10.3) | 9.9 (8.6-11.1) | 9.4 (7.4-11.4) | 9.9 (7.4-12.5) | 10.3 (9.3-11.3) | 7.9 (5.9-10.0) |
| Mexican mixed dishes | 7.3 (5.9-8.8) | 6.8 (5.2-8.5) | 8.3 (6.8-9.9) | 7.3 (5.6-8.9) | 6.0 (4.1-7.8) | 11.0 (7.8-14.3) | 7.1 (5.6-8.6) | 5.2 (3.6-6.7)** |
| Pork | 6.1 (5.1-7.2) | 6.4 (5.2-7.6) | 7.6 (6.2-9.0) | 7.0 (5.1-9.0) | 4.1 (3.0-5.3)*** | 7.2 (5.4-9.0) | 6.4 (5.4-7.5) | 5.7 (3.9-7.4) |
| Grain-based mixed dishes | 4.8 (3.9-5.7) | 6.5 (5.4-7.7) | 6.3 (4.6-8.0) | 5.3 (4.1-6.5) | 5.6 (3.7-7.4) | 5.5 (3.7-7.4) | 5.5 (4.4-6.6) | 6.0 (4.3-7.8) |
| Bacon | 4.0 (3.0-5.0) | 6.5 (5.2-7.8)** | 3.8 (3.0-4.7) | 5.5 (4.2-6.7) | 6.2 (4.5-7.9) | 3.4 (2.0-4.7) | 5.2 (4.0-6.4) | 5.9 (4.2-7.6) |
| Pizza | 5.0 (4.2-5.9) | 4.3 (3.4-5.2) | 4.7 (3.7-5.7) | 5.0 (3.8-6.1) | 4.6 (3.2-6.0) | 4.3 (3.1-5.5) | 4.5 (3.7-5.4) | 5.3 (3.4-7.2) |
| *Unprocessed red meat* | | | | | | | | |
| Burgers | 21.0 (18.9-23.2) | 17.6 (15.4-19.8) | 21.7 (19.1-24.3) | 19.2 (11.9-15.9) | 19.1 (15.7-22.5) | 18.0 (15.1-20.9) | 21.9 (19.5-24.2) | 14.8 (11.0-18.5) |
| Beef | 18.3 (15.2-21.5) | 15.6 (13.3-17.9) | 13.9 (11.9-15.9) | 16.3 (13.2-19.4) | 19.6 (15.8-23.5) | 16.2 (13.0-19.5) | 16.2 (14.2-18.2) | 19.5 (14.1-24.8) |
| Meat mixed dishes | 14.8 (12.5-17.0) | 19.4 (17.4-21.5) | 15.7 (13.6-17.7) | 17.4 (14.5-20.3) | 17.5 (14.2-20.8) | 13.1 (10.4-15.8) | 16.9 (15.0-18.7) | 18.9 (15.1-22.6) |
| Mexican mixed dishes | 12.7 (10.3-15.2) | 12.4 (9.6-15.2) | 14.2 (11.2-17.2) | 13.4 (10.3-16.4) | 10.6 (7.4-13.9) | 19.6 (14.1-25.1) | 12.3 (9.9-14.6) | 9.7 (7.2-12.2)** |
| Pork | 10.1 (8.3-11.8) | 11.1 (9.1-13.0) | 12.5 (10.3-14.7) | 11.5 (8.7-14.3) | 7.4 (5.4-9.4)*** | 11.0 (8.5-13.4) | 10.8 (9.0-12.7) | 9.8 (7.1-12.4) |
| Grain-based mixed dishes | 8.0 (6.5-9.4) | 10.5 (8.7-12.3) | 9.5 (6.9-12.1) | 9.5 (7.5-11.4) | 9.2 (6.1-12.4) | 7.9 (5.0-10.8) | 9.0 (7.4-10.6) | 10.2 (7.2-13.3) |
| *Processed meat* | | | | | | | | |
| Cold cuts and cured meats | 37.4 (34.3-40.6) | 38.0 (34.2-41.9) | 37.1 (32.9-41.4) | 40.8 (37.1-44.5) | 35.8 (31.2-40.5) | 35.9 (30.6-41.2) | 37.6 (33.7-41.4) | 38.7 (33.4-44.1) |
| Sausages and frankfurters | 21.5 (19.1-23.9) | 18.9 (16.5-21.2) | 20.3 (17.6-22.9) | 21.3 (19.1-23.6) | 18.5 (14.8-22.2) | 23.3 (18.6-27.9) | 21.3 (19.4-23.3) | 16.9 (13.0-20.7) |
| Bacon | 12.0 (10.0-14.0) | 16.3 (14.3-18.4) *** | 11.5 (9.5-13.5) | 14.1 (11.6-16.6) | 16.7 (13.4-19.9) | 9.2 (6.3-12.1) | 14.5 (12.5-16.5) | 14.9 (11.6-18.2) |
| Pizza | 10.6 (8.9-12.2) | 9.6 (7.3-12.0) | 11.0 (9.3-12.8) | 9.7 (7.3-12.1) | 10.0 (7.1-12.8) | 9.8 (7.3-12.3) | 10.1 (8.1-12.1) | 10.3 (7.0-13.6) |
| ^1^ Values are survey-weighted mean percent (95% confidence interval) of total grams consumed.  ^2^ Family income was categorized as low (Poverty Income Ratio [PIR] 0–185%), middle (PIR 185–400%), and high (PIR ≥400%).  ^3^ Education was defined as low (Less than high school); medium (High school graduate/GED or equivalent or Some college/Associate degree); and high (College graduate or above). Education categories for teens were defined using the household reference person’s educational attainment.  *p<0.05, **p<0.01, ***p<0.001 for the difference in proportional contribution to meat intake. | | | | | | | | |
